# Zika Virus Focuses the Gain-of-Function Debate

**DOI:** 10.1128/mSphere.00069-16

**Published:** 2016-04-06

**Authors:** Michael J. Imperiale, Arturo Casadevall

**Affiliations:** a Department of Microbiology and Immunology, University of Michigan, Ann Arbor, Michigan, USA; b Department of Molecular Microbiology and Immunology, Johns Hopkins Bloomberg School of Public Health, Baltimore, Maryland, USA

## EDITORIAL

This year, the National Science Advisory Board for Biosecurity (NSABB) will be making recommendations to the U.S. Government regarding the ongoing saga of gain-of-function (GOF) experiments with highly infectious respiratory pathogens, such as influenza virus, severe acute respiratory syndrome (SARS) coronavirus, and Middle East respiratory syndrome (MERS) coronavirus. If adopted into policy by the Government, these recommendations have the potential to alter the way in which research with so-called pathogens with pandemic potential (PPP) is conducted for years to come.

To summarize briefly, the debate about PPP began with the publication of two papers in 2012 that demonstrated the ability to passage highly pathogenic H5N1 avian influenza virus through ferrets and obtain viruses that could be transmitted between the ferrets by an airborne route. In the intervening years, there was continued work to understand the relationship between influenza virulence and transmissibility using ferrets to select for variants with those traits. The impetus for these experiments was a growing number of human cases of H5N1 coupled with the fear that, if the virus acquired the ability to spread directly from human to human and maintained its high mortality rate (or even something within an order of magnitude of it), the consequences of a potential pandemic would be significant. These experiments have conclusively established that H5N1 influenza viruses have the potential for airborne mammalian transmission in ferrets. Thus, if one accepts the widely accepted premise that ferrets are useful experimental surrogates for human influenza transmission, these experiments established that H5N1 has pandemic potential and alerted humanity to this danger. After these papers were published, along with similar reports about related avian influenza strains, calls went out for a moratorium or, in some cases, a complete halt on this type of research (1, 2). The major concern was that a deliberate or accidental release of these new strains from the laboratories could cause a human-made pandemic. As a result of much public back-and-forth between advocates and opponents of GOF research, the U.S. Government imposed a moratorium on certain experiments in late 2014 (3) and asked the NSABB to provide its advice (for a prior summary of this controversy, see reference 4). The National Academy of Sciences was enlisted to convene two workshops, one at the beginning of the NSABB deliberations and one nearer to the end, at which experts in the area provided their insights into the process. Moreover, the NSABB commissioned a report on how a risk-benefit analysis of GOF research might be undertaken.

The first NAS workshop, held in December 2014, was by and large a useful restatement of the various viewpoints that had been making their way through the scientific, lay, and social media. A summary of the workshop was produced and released in early 2015 (5). In the meantime, the NSABB appointed a working group to begin analyzing the issue; this group’s recommendations were formalized by the entire Board in May 2015 (6).

The task of researching the various approaches to performing a risk-benefit analysis was contracted to Gryphon Scientific, a company that consults on biosecurity and other life science-related policy issues. Their report, which weighed in at over 1,000 pages, begins with a thorough summary of the GOF landscape and subsequently presents various risk and benefit scenarios for MERS, SARS, and three categories of influenza, seasonal, pandemic, and avian (7). We encourage the reader to peruse the executive summary for more detail, but it is our impression that this report is both balanced and comprehensive. This report, along with a paper discussing the ethical considerations of GOF research that was also commissioned by the NSABB (8), was discussed at a meeting at the NIH at the beginning of January 2016. The Board heard from a variety of individuals, some of whom have been involved in this debate for many years and others of whom provided fresh voices. Both sides found parts of the Gryphon report that they liked and parts that they disliked. To us, the fact that neither the pro- nor the anti-GOF advocates are completely satisfied with the report means that it struck a good balance. We have previously noted that the major difficulty in resolving this debate is that it is fundamentally a conflict between different philosophies, risk assessments, and value systems and thus cannot be settled on the basis of some objective criteria. The voluminous aspect of the Gryphon report seems to reflect a desire to cover all those subjects for which there are some data that can be analyzed with existing risk-benefit tools. However, the areas that separate pro- and anti-GOF advocates fall into areas of judgment and belief, and these differences cannot be adjudicated by risk-benefit analysis. Apart from providing an assessment of the situation by a disinterested third party, the Gryphon analysis was also helpful in that it bought time for passions to settle and perhaps allow for a more cool-headed conversation that was difficult 2 years ago, when both sides engaged in an acrimonious debate in both the scientific and the general media (4).

As we approach a time of decision, it is critically important that the NSABB makes clear, sensible recommendations to the U.S. Government. While there were relatively few new ideas presented at the January meeting other than those from ethicists, many of the speakers implored the Board to be precise. For example, the Board was asked to provide specific examples of experiments that should or should not be performed, and the members appeared to be receptive to these pleas to provide more clarity than current guidance documents present. In addition, three of the more vocal critics of GOF research with PPP recently proposed six specific policy options for consideration at the second NAS meeting (9), which was held in early March 2016.

A question in our minds is how long it will take the Government to incorporate those recommendations, as it sees fit of course, into policy. There is reason for concern. We note that the original NSABB *Proposed Framework for the Oversight of Dual Use Life Sciences Research* (10) was approved by the Board in June 2007, yet no policy was forthcoming until the H5N1 publications forced the issue almost 5 years later. During that time, no experiments were on hold, as is the case with the current moratorium. Will the fact that we are in a presidential election year mean a *de facto* pause in the policy-making process? The longer the moratorium persists, the more likely it is that important work will not be performed, or perhaps it will simply move overseas with funding from non-U.S. agencies as researchers tire of endless waiting for clear guidance. In fact, GOF work is occurring outside the United States, and several publications describing experiments that are likely to be prohibited by the current moratorium have appeared (e.g., see references [Bibr B11][Bibr B12][Bibr B14]). Hence, the U.S. moratorium is not preventing this type of work. Our concern about moratoriums and the continuing controversy is that it will discourage the best and brightest from working on dangerous pathogens that threaten humanity. In this regard, there is preliminary evidence that the controversy is being closely followed by younger scientists and that it may be affecting their choice of research careers (15).

As the great GOF debate lumbers on without resolution, a new infectious threat has suddenly appeared in the form of Zika virus. Zika virus was discovered in a sentinel monkey in 1974 in the Zika forest in Uganda. Over the next 3 decades, it caused only a few documented human cases. However, in recent years, it spread to the Pacific islands, and we are now in the midst of a serious epidemic in South America. Although Zika virus infection appears to cause a self-limited disease in most individuals, there are ominous reports of an association with microcephaly in babies of affected mothers and neurological disorders, such as Guillain-Barre, in some affected adults. At the time of this writing, these associations have not been proven causative, and it is unclear whether these complications are new facets of Zika virus disease as a result of increased pathogenicity by passage in the human population or a new realization of rare complications as the virus infects millions.

The Zika virus provides a new lens with which to view the great GOF debate. This serves as a reminder to us of why proponents of GOF argue that work on dangerous pathogens should continue and why opponents of GOF argue for a halt because of the threat of laboratory-derived pandemics. GOF types of experiments could be used to ascertain whether the potential for causing neuropathology and microcephaly is a potential characteristic of this virus that has finally emerged by recent passage in human populations. For example, comparison of earlier and current Zika virus isolates might reveal differences that could be associated with new symptoms. Showing that the virus can acquire those new properties through GOF-type experiments would help establish causality and might provide new insights for therapy and vaccines. As we have noted, GOF-type experiments are epistemologically rich and can provide unambiguous answers to problems of causality (16). Hence, we have argued that GOF experiments are powerful tools of human inquiry that should have a role in studying problems of virulence and transmissibility, provided that these studies can be performed safely. On the other hand, the Zika virus outbreak is an example of what concerns the anti-GOF proponents, the rapid spread of a new virus in human populations with the potential to cause devastating damage in those populations. Fortunately, it does not appear that Zika virus can spread by a respiratory route, which undoubtedly would make control much more difficult to achieve. Although the Zika virus outbreak does not help to resolve the GOF debate, it does provide a lens that helps to refine the resolution of both pro- and anti-GOF arguments.

The threat of viral pandemics and other infectious diseases is ever-present. It is obvious that our best defense against this threat is advancing our knowledge such that we understand the biology of the pathogens and what determines their virulence in human and animal hosts. These studies, when undertaken in a safe and responsible manner, will inform the development of diagnostics, vaccines, and therapeutics. It is essential that the moratorium end as soon as possible, before nature outguns us.
